# Monitoring of First-line Drug Resistance Mutations Outside the Scope of Xpert MTB/RIF Ultra is Needed for Successful Control of DR-TB in Southern Mozambique

**DOI:** 10.1093/cid/ciad684

**Published:** 2023-12-04

**Authors:** Carla Mariner-Llicer, Belén Saavedra Cervera, Edson Mambuque, Neide Gomes, Shilzia Munguambe, Luis Villamayor, Irving Cancino-Muñoz, Manuela Torres-Puente, Dinis Nguenha, Durval Respeito, Gustavo Tembe, Mariana G López, Iñaki Comas, Alberto L García-Basteiro

**Affiliations:** Tuberculosis Genomics Unit, Instituto de Biomedicina de Valencia (IBV), CSIC, Valencia, Spain; ISGlobal, Hospital Clínic – Universitat de Barcelona, Barcelona, Spain; Centro de Investigação em Saúde de Manhiça (CISM), Maputo, Mozambique; Centro de Investigação em Saúde de Manhiça (CISM), Maputo, Mozambique; Centro de Investigação em Saúde de Manhiça (CISM), Maputo, Mozambique; Centro de Investigação em Saúde de Manhiça (CISM), Maputo, Mozambique; FISABIO Public Health, Valencia, Spain; FISABIO Public Health, Valencia, Spain; I2SysBio, Universitat de València CSIC, Valencia, Spain; Tuberculosis Genomics Unit, Instituto de Biomedicina de Valencia (IBV), CSIC, Valencia, Spain; Centro de Investigação em Saúde de Manhiça (CISM), Maputo, Mozambique; Amsterdam Institute for Global Health & Development (AIGHD), Amsterdam, The Netherlands; Centro de Investigação em Saúde de Manhiça (CISM), Maputo, Mozambique; Centro de Investigação em Saúde de Manhiça (CISM), Maputo, Mozambique; Tuberculosis Genomics Unit, Instituto de Biomedicina de Valencia (IBV), CSIC, Valencia, Spain; Tuberculosis Genomics Unit, Instituto de Biomedicina de Valencia (IBV), CSIC, Valencia, Spain; CIBER in Epidemiology and Public Health, Madrid, Spain; ISGlobal, Hospital Clínic – Universitat de Barcelona, Barcelona, Spain; Centro de Investigação em Saúde de Manhiça (CISM), Maputo, Mozambique; Centro de Investigación Biomédica en Red de Enfermedades Infecciosas (CIBERINFEC), Barcelona, Spain

**Keywords:** *Mycobacterium tuberculosis*, whole-genome sequencing, resistance conferring mutations, Mozambique

## Abstract

Multidrug-resistant(MDR) tuberculosis in Southern Africa is of great concern, exacerbated by the spread of a clone harboring a mutation missed by Xpert Ultra. In Southern Mozambique, the presence of such mutation and rising cases of non-MDR isoniazid resistance highlights the need to ensure accurate detection of antimicrobial-resistance in the country.

Mozambique is among the 30 countries with the highest burden of multidrug-resistant tuberculosis (MDR-TB) according to the World Health Organization (WHO). In 2021, 3.5% and 11% MDR-TB cases were reported among new and previously treated patients respectively, matching the last national estimates of 2008 [1]. Recently, they have implemented Xpert MTB/RIF Ultra (XpertUltra), a WHO-endorsed rapid method that identifies *Mycobacterium tuberculosis* complex (MTBC) and rifampicin-resistance (RIF-R) (WHO treatment guidelines, 2020). This assay detects well-characterized mutations in the RIF-R determining region (RRDR) of *rpoB*, an 81-bp “core region” where the most common mutations, responsible for 95%–97% of RIF-R cases, are located [2]. Currently, the monitoring of RIF-R using XpertUltra has been challenged in southern Africa due to the spread of an MDR-TB clone harboring a low-level resistance mutation, *rpoB_*I4941F, which is missed by XpertUltra and also by phenotypic DST (pDST) [3, 4]. This mutation was reported in Eswatini for the first time in 2015, where it had been silently transmitted since 2009 causing an MDR-TB outbreak [4]. Later, 2 clones bearing the variant were detected in South Africa (2013–2016), one introduced from Eswatini and the other likely emerging independently [3]. Additionally, the MDR prevalence in both countries is high with 5% and 14% among new and retreated cases in Eswatini; and 4.1% and 28% in South Africa (WHO). The latest antimicrobial resistance (AMR) prevalence estimates for southern Mozambique, obtained through pDST in 2014, showed an MDR-TB profile comparable to the latest national estimates (3.8% new and 13.2% retreated cases) [1, 5]. Nevertheless, these MDR prevalence rates are likely underestimated, as some RIF-R mutations missed by XpertUltra are also undetected by pDST, as they cause bacterial growth retardation [6]. In this regard, a comprehensive screening for AMR-conferring mutations is essential to develop targeted public health strategies to improve diagnosis and treatment of drug-resistant TB (DR-TB).

Given the concerning MDR figures in Mozambique's bordering countries together with the high level of migration, particularly to South Africa, it is imperative to assess the presence and spread of *rpoB*_I491F and additional mutations missed by XpertUltra, including those associated to isoniazid, especially in the southern border region. The aim of this study is to screen the AMR-conferring mutations, focusing mainly on those located outside the RRDR, and to reassess the current AMR situation in Southern Mozambique.

## METHODS

This study was conducted in the district of Manhiça, Maputo province, a rural area with high TB/human immunodeficiency virus (HIV) burden and TB notification rate in southern Mozambique [7]. Manhiça is close to the South Africa and Eswatini borders and reports a high mobility rate with both countries [8]. The samples included in this study are from 2 different surveys. (i) 337 TB culture-positive from an index-contacts study (January–December 2018). (ii) 275 publicly available sequences obtained from a prospective surveillance-based study (August 2013 to 2014) [9]. All isolates were whole-genome sequenced (WGS) by short-read sequencing approach (Illumina) and analyzed running our published and validated routine pipeline (https://gitlab.com/tbgenomicsunit/ThePipeline). We looked for all the AMR-associated single-nucleotide polymorphisms (SNPs) and indels listed in the catalog [10]. We took a closer look by performing an expert-knowledge analysis to find polymorphisms in candidate genes conferring resistance to bedaquiline (BDQ) and delamanid (DLM) [11]. We also searched for frameshift and insertion elements in the *Rv0678* gene as they have been associated to increase the value of the minimum inhibitory concentration for BDQ. Furthermore, we calculated clustering by pairwise distance to identify recent (5 SNPs) local transmission [9] and quantify transmission events. Additionally, we downloaded all 10 000 available WGS African sequences from the ENA repository to identify any potential transmission link (10 SNPs) (Supplementary Table 1**)**. Analysis was performed following the same pipeline described above. We constructed an alignment of high confidence fixed-SNPs (fSNPs, >90% frequency), and obtained transmission clusters (Supplementary Material 1).

## RESULTS

We identified 78/612 (12.7%) strains containing at least 1 AMR-conferring mutation: 38 (6.2%) mono-resistant, 21 (3.4%) poly-resistant, 17 (2.8%) MDR-TB, and 2 (0.3%) pre-extensively resistant (pre-XDR, according to 2021 WHO definition) (Supplementary Table 2). Regarding the 2 most common first-line drugs, we found 59 (9.6%) isoniazid resistant (INH-R) and 25 (4.1%) RIF-R strains. The most prevalent mutations were: *katG*_S315T in 31/59 (53%) INH-R strains; *inhA*_c-777t (*fabG1*_c-15t), conferring cross-resistance to INH and ethionamide (ETH), in 20/59 (34%) INH-R strains; *rpoB*_S450L in 15/25 (63%) RIF-R strains. We identified 2 additional RIF-R conferring mutations located outside the RRDR region and undetected by XpertUltra: *rpoB*_V170F (fSNP) in 2 strains from 2014; and *rpoB*_I491F (6.5% frequency) in 1 strain from 2018. These strains had no additional AMR mutations. Furthermore, we detected 2 mutations in the RRDR region, usually missed by pDST [6, 12]: *rpoB*_D435F (81% frequency) in 1 MDR-TB strain harboring another variant (*rpoB*_S450L, fSNP); and *rpoB*_H445N at 6.6% frequency in 1 strain with no additional variants but fixed in another MDR-TB strain. Regarding second-line drugs, we detected 2 mutations (*gyrA_*D94G, *gyrA_*D94A) conferring resistance to levofloxacin and moxifloxacin in 2 pre-XDR strains. Importantly, we did not find variants in known genes associated with BDQ and DLM resistance, or truncations in *Rv0678* (Table 1).

**Table 1.** AMR-conferring Mutations Found in Mozambican Strains (N = 612)

Antibiotic	Strains With Resistance Mutations (%)	Gene	Mutation	2018	2014	Total
INH	38 (6.2)	*katG*	S315T	22	9	31
		*katG*	katG_1285_del_1_gc_g	0	3	3
		*katG*	katG_1433_del_1_gc_g	2	0	2
		*katG*	katG_1866_del_1_ag_a	0	1	1
INH, ETH	25 (4.1)	*IG_Rv1482c_Rv1483*	inhA_c-777t (fabG1_c-15t)	11	9	20
		*fabG1-inhA*	L203L	3	0	3
		*IG_Rv1482c_Rv1483*	inhA_t-770a (fabG1_t-8a)	0	2	2
RIF	25 (4.1)	*rpoB*	S450L	9	6	15
		*rpoB*	H445D	2	0	2
		*rpoB*	H445N	1	1	2
		*rpoB*	S450Y	2	0	2
		*rpoB*	V170F^a^	2	0	2
		*rpoB*	D435F^b^	1	0	1
		*rpoB*	H445Y	0	1	1
		*rpoB*	I491F^a^	0	1	1
EMB	14 (2.3)	*embB*	M306I	4	4	8
		*embB*	G406D	2	0	2
		*embB*	M306L	2	0	2
		*embB*	D354A	1	0	1
		*embB*	M306V	1	0	1
ETH	5 (0.8)	*ethA*	ethA_33_del_1_gc_g	2	0	2
		*ethA*	ethA_861_del_1_gt_g	2	0	2
		*inhA*	S94A	0	1	1
PZA	8 (1.3)	*pncA*	pncA_390_del_1_ca_c	0	2	2
		*pncA*	T135P	2	0	2
		*pncA*	C14R	1	0	1
		*pncA*	L27P	0	1	1
		*pncA*	V130G^c^	0	1	1
		*pncA*	V139G	1	0	1
		*pncA*	Y34D	0	1	1
SM	25 (4.1)^d^	*rpsL*	K43R	8	2	10
		*IG_Rv1315_Rv1316c*	rrs_a1401g	0	5	5
		*gid*	gid_116_del_1_cg_c	4	0	4
		*gid*	gid_103_del_1_gc_g	0	3	3
		*IG_Rv1315_Rv1316c*	rrs_a514c	1	1	2
		*gid*	gid_352_del_1_gc_g	1	0	1
		*gid*	gid_472_del_1_gc_g	1	0	1
LEV, MXF	2 (0.3)	*gyrA*	D94A	1	0	1
		*gyrA*	D94G	1	0	1
AMI, CAP, KAN	1 (0.2)	*IG_Rv1315_Rv1316c*	rrs_a1401g	1	0	1
CAP	1 (0.2)	*tlyA*	Q22!	0	1	1

Abbreviations: AMI, amikacin; AMR, antimicrobial resistance; CAP, capreomycin; EMB, ethambutol; ETH, ethionamide; INH, isoniazid; LEV, levofloxacin; MXF, moxifloxacin; PZA, pyrazinamide; RIF, rifampicin; SM, streptomycin;

^a^Represents mutations outside the RRDR region and, therefore, undetected by XpertUltra.

^b^rpoB_D435F appears in one strain with rpoB_S450L.

^c^pncA_V130G appears in the same strain with pncA_390_del_1_ca_c.

^d^One strain SM-R harbors 2 mutations: rrs_a1401g and rpsL_K43R.

Regarding local transmission, we overall identified 190 putative transmission events among Mozambican cases, 28 of them occurring between AMR strains (14.7%, 28/190); this proportion was lower than transmission events occurring between susceptible strains (85.3%, 162/190) (odds ratio = 1.42; 95% confidence interval [CI]: .83–2.44; *P* value = .2). We also characterized transmission of the most prevalent mutations to first-line drugs: 19/31 strains with *katG*_S315T were within 6 different clusters; and 4/15 strains with *rpoB*_S450L were within 2 transmission clusters.

Through the transmission analysis between Mozambique and other African countries, we identified 30 intercountry clusters involving at least 1 Mozambican strain and 1 or more from others. Clusters involved 52 Mozambican samples and 150 from surrounding countries, mainly South Africa (73) and Eswatini (40) but also from Botswana, Malawi, and Zimbabwe; with a cluster size range of 2–35 samples. Importantly, 5/30 (16.7%) clusters presented a mean distance below 5 SNPs (range 1–5) suggesting very recent cross-border transmission.

## DISCUSSION

Here we presented a WGS screening of drug-resistance conferring mutations in Mozambique. The *rpoB*_I491F mutation was detected (not fixed) in 1 case from 2014, which was not in cluster with other samples. The strain did not present a polyclonal infection suggesting that the mutation arose de novo. Furthermore, we detected 2 strains from 2018 harboring *rpoB*_V170F mutation, previously reported in the country [13]. As we detected borderline resistance mutations in the RRDR, which are easily missed by pDST, it is likely that a proportion of RIF resistant patients are being misdiagnosed and receiving ineffective treatment. Therefore, increasing the risk of transmission of AMR strains and negative outcomes as have been shown [12].

We have not detected a spread of *rpoB*_I491F from neighboring countries to Southern Mozambique although it has been circulating widely in South Africa and Eswatini since 2009 [3, 4]. However, we show evidence of transmission between countries for other strains even though our transmission analysis is limited in time and not nationwide. This, together with the high rate of cross-border mobility, alerts the need of increasing surveillance of AMR strains.

We found a prevalence of DR-TB of 2.8% (2018), slightly lower than the obtained in the last pDST-based survey (3.8%) conducted in Manhiça (2014) [5], suggesting a broadly stable situation compared to neighboring countries [3, 4]. We detected an increasing trend in new INH-R cases, 10.9%, compared to 2014 (9.2%) [5] driven by a higher percentage of INH-R non-MDR strains (7.6% in 2018 vs 5.5% in 2014). The results suggest that almost 1 in 10 cases by 2018 were INH-R before starting treatment and undetected by XpertUltra. High-burden TB countries have limited tools to detect INH-R cases as they tend to be centralized in reference laboratories, diagnosing and treating them as pansusceptible [14]. Close monitoring of AMR is therefore essential to guarantee that the currently recommended standardized regimens for treating DR-TB continue to be effective.

A limitation of this analysis is that the estimates cannot be extrapolated to the whole country, as the strains included to identify AMR-conferring mutations were collected in Manhiça district. In addition, we cannot assess the correlation with pDST data. However, the aim of the study was to identify circulation and prevalence of mutations rather than concordance with pDST. Opportunely, the accuracy of WGS for first-line AMR prediction shows a high sensitivity and specificity, (>90%), for RIF and INH making both techniques comparable [10].

Our results show the most recent AMR figures in southern Mozambique. Although much attention must be paid to RIF-R, especially by expanding the AMR-targets beyond those implemented in XpertUltra, INH-R is now recognized as another major challenge for TB control and successful treatment outcomes [14]. The genomic evidence provided here, together with the MDR situation in the region, call for a thoughtful choice of surveillance tools and diagnostic algorithms. It is thus needed to assess the implementation of new molecular tests, such as Xpert MTB/XDR, low complexity automated nucleic acid amplification tests (NAAT) or targeted sequencing as rapid approaches for the detection of both INH and RIF-resistance, thereby simultaneously interrogating a larger number of well-characterized mutations [10] and providing a more reliable drug-resistance profile.

## Supplementary Data

Supplementary materials are available at *Clinical Infectious Diseases* online. Consisting of data provided by the authors to benefit the reader, the posted materials are not copyedited and are the sole responsibility of the authors, so questions or comments should be addressed to the corresponding author.

## Supplementary Material

ciad684_Supplementary_Data

## Notes

***Author Contributions***. I. C., A. G. B., and M. G. L. have contributed to the conception and the design of the work. E. M., S. M., N. G., L. V., I. C. M., M. T. P., D. N., D. D. R., and G. T. have processed the samples and acquired the epidemiological data that have been analyzed by B. S. C. and C. M. L. Interpretation of results has been made by C. M. L., M. G. L., I. C., A. G. B. and B. S. C. C. M. L. and M. G. L. have drafted the manuscript and I. C., B. S. C., and A. G. B. have contributed to its revision. All authors have agreed to final approval for submission.

***Acknowledgments.*** The authors would like to express our gratitude to Stefan Niemann (Research Center Borstel Leibniz Lung Center, Borstel, Germany) and to Alex Rosenthal (Office of Cyber Infrastructure and Computational Biology, National Institutes of Health, Rockville, Maryland, USA) from TB PORTALS for performing and funding the sequencing of the samples included in this study.

***Ethical statement.*** The study was approved by the National Bioethics Committee for Health of Mozambique (CNBS, Ref:369/CNBS/17) and the Internal Bioethics Committee of CISM. All methods were performed in accordance with the relevant guidelines and regulations. All study participants signed written informed consent after a verbal explanation and written information about the study was provided. For participants under 18 years old, informed consent was obtained from their relatives, parents, or guardians.

***Data availability***. Sequences generated in this study (study no. 1) were uploaded to the European Nucleotide Archive (ENA) under the accession number PRJEB61426. Sequences from study no. 2 are available under the accession numbers: PRJEB27421, PRJEB50708 and PRJEB49677. Study codes from the African data sets are detailed in S2. Supplementary Material Table 1.

***Financial support*.** This work was supported by STOP TB partnership (STBP/TBREACH/GSA/W5-30: CA-3-D000920001 to AGB), the TB Portals program of the National Institutes of Health, the European Research Council (TB RECONNECT: H2020-ERC-COG/0800 to I. C.,), Generalitat Valenciana (AICO/2018/113 to I. C.) and the Spanish Ministry of Science and Innovation (PID2019-104477RB-I00 to I. C.). The funders had no role in study design, data collection and interpretation, or the decision to submit the work for publication.

## References

[1] Samo Gudo P, Cuna Z, Coelho E, et al Is multidrug-resistant tuberculosis on the rise in Mozambique? Results of a national drug resistance survey. Eur Respir J 2011; 38:222–4. 21719501 10.1183/09031936.00182010

[2] Chakravorty S, Simmons AM, Rowneki M, et al The new Xpert MTB/RIF Ultra: improving detection of *Mycobacterium tuberculosis* and resistance to rifampin in an assay suitable for point-of-care testing. MBio 2017; 8:e00812-17. 28851844 10.1128/mBio.00812-17 PMC5574709

[3] Makhado NA, Matabane E, Faccin M, et al Outbreak of multidrug-resistant tuberculosis in South Africa undetected by WHO-endorsed commercial tests: an observational study. Lancet Infect Dis 2018; 18:1350–9. 30342828 10.1016/S1473-3099(18)30496-1

[4] Beckert P, Sanchez-Padilla E, Merker M, et al MDR *M. tuberculosis* outbreak clone in Eswatini missed by Xpert has elevated bedaquiline resistance dated to the pre-treatment era. Genome Med 2020; 12:104. 33239092 10.1186/s13073-020-00793-8 PMC7687760

[5] Valencia S, Respeito D, Blanco S, et al Tuberculosis drug resistance in Southern Mozambique: results of a population-level survey in the district of Manhiça. Int J Tuberc Lung Dis 2017; 21:446–51. 28284261 10.5588/ijtld.16.0694

[6] Miotto P, Cabibbe AM, Borroni E, Degano M, Cirillo DM. Role of disputed mutations in the *rpoB* gene in interpretation of automated liquid MGIT culture results for rifampin susceptibility testing of *Mycobacterium tuberculosis*. J Clin Microbiol 2018; 56:e01599-17. 29540456 10.1128/JCM.01599-17 PMC5925711

[7] García-Basteiro AL, Miranda Ribeiro R, Brew J, et al Tuberculosis on the rise in southern Mozambique (1997–2012). Eur Respir J 2017; 49:1601683. 28331035 10.1183/13993003.01683-2016

[8] Bernardo EL, Nhampossa T, Clouse K, et al Patterns of mobility and its impact on retention in care among people living with HIV in the Manhiça District, Mozambique. PLoS One 2021; 16:e0250844. 34019556 10.1371/journal.pone.0250844 PMC8139482

[9] Saavedra Cervera B, López MG, Chiner-Oms Á, et al Fine-grain population structure and transmission patterns of *Mycobacterium tuberculosis* in southern Mozambique, a high TB/HIV burden area. Microb Genom 2022; 8:mgen000844. 10.1099/mgen.0.000844 PMC9455694 35787782

[10] Walker TM, Miotto P, Köser CU, et al The 2021 WHO catalogue of *Mycobacterium tuberculosis* complex mutations associated with drug resistance: a genotypic analysis. Lancet Microbe 2022; 3:e265–73. 35373160 10.1016/S2666-5247(21)00301-3 PMC7612554

[11] Kadura S, King N, Nakhoul M, et al Systematic review of mutations associated with resistance to the new and repurposed *Mycobacterium tuberculosis* drugs bedaquiline, clofazimine, linezolid, delamanid and pretomanid. J Antimicrob Chemother 2020; 75:2031–43. 32361756 10.1093/jac/dkaa136 PMC7825472

[12] Van Deun A, Decroo T, Aung KJM, et al *Mycobacterium tuberculosis* borderline *rpoB* mutations: emerging from the unknown. Eur Respir J 2021; 58:2100783. 33926970 10.1183/13993003.00783-2021

[13] Feliciano CS, Namburete EI, Rodrigues Plaça J, et al Accuracy of whole genome sequencing versus phenotypic (MGIT) and commercial molecular tests for detection of drug-resistant *Mycobacterium tuberculosis* isolated from patients in Brazil and Mozambique. Tuberculosis 2018; 110:59–67. 29779775 10.1016/j.tube.2018.04.003 PMC9328935

[14] Sulis G, Pai M. Isoniazid-resistant tuberculosis: a problem we can no longer ignore. PLoS Med 2020; 17:e1003023. 31961857 10.1371/journal.pmed.1003023 PMC6974036

