# Supplementary material for: Monitoring of First-line Drug Resistance Mutations Outside the Scope of Xpert MTB/RIF Ultra is Needed for Successful Control of DR-TB in Southern Mozambique
Source: Clin Infect Dis. 2023 Dec 4;78(4):842–5. doi: 10.1093/cid/ciad684 (PMC11006097; doi:10.1093/cid/ciad684)
Supplement: ciad684_Supplementary_Data [file ciad684_supplementary_data.pdf]

SUPPLEMENTARY MATERIAL

Title:

Monitoring of first-line drug resistance mutations outside the scope of Xpert MTB/RIF Ultra is needed for successful control of DR-TB in Southern Mozambique

S1. Supplementary Methods

This study was conducted in the district of Manhiça, Maputo province, a rural area with high TB/HIV burden and TB notification rate in southern Mozambique [1]. Manhiça is close to the South Africa and Eswatini borders and reports a high mobility rate with both neighbouring countries [2]. The Centro de Investigação em Saúde de Manhiça (CISM), receives samples from several point-of-care centres throughout the province.

Quality thresholds used for excluding sequences for downstream analysis were: median depth < 50X and genome coverage < 90%. We screen for all variants above 5% frequency to identify all the AMR-associated SNPs and indels listed in the WHO catalogue.

Regarding second-line drugs, we performed an expert-knowledge based analysis to find polymorphisms in candidate genes conferring resistance to bedaquiline (BDQ) (*atpE, Rv0678* and *pepQ*) and delamanid (DLM) (*ddn, fgd1, fbiA, fbiB, fbiC* and *fbiD*). To do that, we look for all the mutations listed in Kadura et al. 2020 [3]. Additionally, we searched for frameshift and insertion elements in *Rv0678* gene, which have been recently associated with increments in the minimum inhibitory concentrations (MICs) for BDQ as detailed in Roberts *et al.* 2022 [4]. Of note, the current version of the WHO catalogue lacks mutations conferring resistance to BDQ and clofazimine (CLZ).

Transmission events within Mozambican strains were defined as the number of cases in cluster minus one. They were measured by taking into account those “unmixed” clusters containing only resistant or only susceptible strains as detailed by Walker *et al.* 2014 [5].

The analysis of tuberculosis transmission within Mozambique and other African countries included sequences from the neighbouring countries Eswatini, South Africa, Tanzania, Malawi, Zimbabwe and Botswana. See **S2. Supplementary Table1** for detailed information of the datasets. Only those sequences meeting the quality criteria mentioned above were included in the analysis. We constructed an alignment of high confidence fixed-SNPs (fSNPs, >90% frequency), measured pairwise distances and obtained transmission clusters at 10 SNPs cut-off.

Supplementary Table S1. Accession codes and amount of downloaded sequences included in the analysis.

Country	Study Accession ID	Doi	Amount of Samples
Algeria	PRJEB41267	CRyPTIC. San Raffaele Scientific Institute. Algeria	81
Botswana	PRJEB62480	10.3201/eid2905.220796	1421
Botswana	PRJNA436223		11
Botswana	PRJNA670836	10.12688/f1000research.28318.1	28
Congo	PRJEB27847	10.1016/j.ebiom.2018.10.013	324
Congo	PRJEB9545	10.3201/eid2303.160679	122
Congo	PRJNA300846	10.1016/S2666-5247(21)00044-6	33
Djibouti	PRJNA393924	10.1038/s41598-017-17705-3	131
Eswatini	PRJEB37777	10.1186/s13073-020-00793-8	248
Eswatini	PRJEB6273		
Eswatini	PRJEB7281		
Eswatini	PRJEB9680		
Ethiopia	PRJEB9201	10.1016/j.cub.2015.10.061	28
Gambia	PRJEB36076	10.1016/j.tube.2020.101899	442
Ghana	PRJNA616081	10.3389/fmed.2020.00161	434
Ghana	PRJEB4884	10.1038/s41598-018-29620-2	192
Ghana	PRJEB9003		
Ghana	PRJEB23179		
Ghana	PRJEB3223		
Ghana	PRJEB9545		
Ghana	PRJEB6273		
Côte d'Ivoire	PRJNA454477	10.1128/AAC.02175-18	43
Kenya	PRJEB50767	10.3390/genes13030475	365
Kenya	PRJNA300846	10.1016/S2666-5247(21)00044-6	15
Liberia	PRJEB32589	10.1099/mgen.0.000325	40
Malawi	PRJEB2358	10.7554/eLife.05166	331
Malawi	PRJEB2794	10.7554/eLife.05166	1877
Mozambique	PRJEB27421	10.1099/mgen.0.000844	275
Mozambique	PRJEB50708		
Mozambique	PRJEB49677		
Mozambique	PRJEB61426	published in this paper	337
Mozambique	PRJEB23648	10.1016/j.tube.2018.04.003	13

Nigeria	PRJEB15857	10.1371/journal.pone.0184510	15
Nigeria	PRJNA300846	IeDEA consortium	12
Rwanda	PRJEB43270	10.1016/j.jctube.2022.100299	419
SouthAfrica	PRJEB14199	10.1016/S2213-2600(16)30433-7	151
SouthAfrica	PRJEB43283	10.1099/mgen.0.000815	359
SouthAfrica	PRJNA183624	10.1371/journal.pmed.1001880	212
SouthAfrica	PRJNA300846	10.1016/S2666-5247(21)00044-6	111
SouthAfrica	PRJNA428596	10.1016/S1473-3099(18)30073-2	1081
SouthAfrica	PRJNA670836	10.12688/f1000research.28318.1	22
SouthAfrica	TBARC	TB-ARC - MRC SA	179
Tanzania	PRJEB49562	10.1371/journal.ppat.1010893	676
Tanzania	PRJNA300846	10.1016/S2666-5247(21)00044-6	29
Tanzania	PRJNA670836	10.12688/f1000research.28318.1	390
Tunisia	PRJEB30463	10.3201/eid2503.181370	46
Tunisia	PRJEB39509	10.1016/j.ijid.2020.11.195	29
Tunisia	PRJNA306588	10.1016/j.ijid.2020.11.195	13
Uganda	PRJEB2424	10.1371/journal.pone.0083012	51
Zimbabwe	PRJEB18529	10.1186/s12916-017-0834-4	26

Supplementary Table S2: Resistance profile of all the strains harbouring at least one mutation included in the WHO Catalog [6].

Patient	INH	RMP	ETH	SM	PZA	EMB	KAN	AMK	CPR	LEV	MXF	BDQ	DLM	mono/poly resistance	poly-R Resistance profile	INH Type
1705225_2	fabG1_inhA_c-777t (fabG1_c-15t)	S	fabG1_inhA_c-777t (fabG1_c-15t)	S	S	S	S	S	S	S	S	S	S	poly-R	INH+ETH	INH_noMDR
1726308_5	fabG1_inhA_c-777t (fabG1_c-15t)	S	fabG1_inhA_c-777t (fabG1_c-15t)	S	S	S	S	S	S	S	S	S	S	poly-R	INH+ETH	INH_noMDR
1693154_1	fabG1_inhA_g-154a (fabG1_L203L)	S	fabG1_inhA_g-154a (fabG1_L203L)	S	S	S	S	S	S	S	S	S	S	poly-R	INH+ETH	INH_noMDR
1689552_2	fabG1_inhA_c-777t (fabG1_c-15t)	S	fabG1_inhA_c-777t (fabG1_c-15t)	S	S	S	S	S	S	S	S	S	S	poly-R	INH+ETH	INH_noMDR
1705053_1	fabG1_inhA_c-777t (fabG1_c-15t)	S	fabG1_inhA_c-777t (fabG1_c-15t)	S	S	S	S	S	S	S	S	S	S	poly-R	INH+ETH	INH_noMDR
1720088_2	fabG1_inhA_c-777t (fabG1_c-15t)	S	fabG1_inhA_c-777t (fabG1_c-15t)	S	S	S	S	S	S	S	S	S	S	poly-R	INH+ETH	INH_noMDR
1758534_7	fabG1_inhA_c-777t (fabG1_c-15t)	S	fabG1_inhA_c-777t (fabG1_c-15t)	S	S	S	S	S	S	S	S	S	S	poly-R	INH+ETH	INH_noMDR
1689410_5	fabG1_inhA_g-154a (fabG1_L203L)	S	fabG1_inhA_g-154a (fabG1_L203L)	S	S	S	S	S	S	S	S	S	S	poly-R	INH+ETH	INH_noMDR
MZ125	fabG1_inhA_c-777t (fabG1_c-15t)	S	fabG1_inhA_c-777t (fabG1_c-15t)	S	S	S	S	S	S	S	S	S	S	poly-R	INH+ETH	INH_noMDR
MZ133	fabG1_inhA_c-777t (fabG1_c-15t)	S	fabG1_inhA_c-777t (fabG1_c-15t)	S	S	S	S	S	S	S	S	S	S	poly-R	INH+ETH	INH_noMDR
MZ146	fabG1_inhA_c-777t (fabG1_c-15t)	S	fabG1_inhA_c-777t (fabG1_c-15t)	S	S	S	S	S	S	S	S	S	S	poly-R	INH+ETH	INH_noMDR

**MZ158**	fabG1_inhA_c-777t (fabG1_c-15t)	S	fabG1_inhA_c-777t (fabG1_c-15t)	S	S	S	S	S	S	S	S	S	S	poly-R	INH+ETH	INH_noMDR
**MZ180**	fabG1_inhA_c-777t (fabG1_c-15t)	S	fabG1_inhA_c-777t (fabG1_c-15t)	S	S	S	S	S	S	S	S	S	S	poly-R	INH+ETH	INH_noMDR
**MZ277**	fabG1_inhA_c-777t (fabG1_c-15t)	S	fabG1_inhA_c-777t (fabG1_c-15t)	S	S	S	S	S	S	S	S	S	S	poly-R	INH+ETH	INH_noMDR
**MZ142**	fabG1_inhA_c-777t (fabG1_c-15t)	S	fabG1_inhA_c-777t (fabG1_c-15t)	S	S	S	S	S	S	S	S	S	S	poly-R	INH+ETH	INH_noMDR
**MZ53**	fabG1_inhA_c-777t (fabG1_c-15t)	S	fabG1_inhA_c-777t (fabG1_c-15t)	gid_103_del_1_gc_g	S	S	S	S	S	S	S	S	S	poly-R	INH+ETH+SM	INH_noMDR
**1688828_9**	katG_S315T	S	S	gid_472_del_1_gc_g	S	S	S	S	S	S	S	S	S	poly-R	INH+SM	INH_noMDR
**MZ271**	katG_S315T	S	S	rpsL_K43R	S	S	S	S	S	S	S	S	S	poly-R	INH+SM	INH_noMDR
**MZ74**	katG_S315T	S	S	gid_103_del_1_gc_g	S	S	S	S	S	S	S	S	S	poly-R	INH+SM	INH_noMDR
**MZ78**	katG_S315T	S	S	gid_103_del_1_gc_g	S	S	S	S	S	S	S	S	S	poly-R	INH+SM	INH_noMDR
**1669572_6**	fabG1_inhA_g-154a (fabG1_L203L)	rpoB_S450L;rpoB_D435F	fabG1_inhA_g-154a (fabG1_L203L)	S	S	S	S	S	S	S	S	S	S	MDR	INH+RMP+ETH	INH_MDR
**1686630_0**	fabG1_inhA_c-777t (fabG1_c-15t)	rpoB_H445D	fabG1_inhA_c-777t (fabG1_c-15t)	rpsL_K43R	S	embB_M306I	S	S	S	S	S	S	S	MDR	INH+RMP+ETH+SM+EMB	INH_MDR
**1686647_8**	katG_S315T	rpoB_S450L	ethA_33_del_1_gc_g	rpsL_K43R	S	embB_M306L	S	S	S	S	S	S	S	MDR	INH+RMP+ETH+SM+EMB	INH_MDR
**1688851_7**	fabG1_inhA_c-777t (fabG1_c-15t)	rpoB_H445D	fabG1_inhA_c-777t (fabG1_c-15t)	rpsL_K43R	S	embB_M306I	S	S	S	S	S	S	S	MDR	INH+RMP+ETH+SM+EMB	INH_MDR
**1705272_6**	katG_S315T	rpoB_S450L	ethA_861_del_1_gt_g	rpsL_K43R	pncA_T135P	embB_D354A	S	S	S	S	S	S	S	MDR	INH+RMP+ETH+SM+PZA+EMB	INH_MDR

1705328_0	fabG1_inhA_c-777t (fabG1_c-15t)	rpoB_H445N	fabG1_inhA_c-777t (fabG1_c-15t)	S	S	S	S	S	S	S	S	S	S	MDR	INH+RMP+ETH	INH_MDR
1720362_3	katG_S315T	rpoB_S450L	ethA_33_del_1_gc_g	rpsL_K43R	S	embB_M306L	S	S	S	S	S	S	S	MDR	INH+RMP+ETH+SM+EMB	INH_MDR
1720507_8	katG_S315T	rpoB_S450L	S	rpsL_K43R	pncA_V139G	embB_M306I	S	S	S	S	S	S	S	MDR	INH+RMP+SM+PZA+EMB	INH_MDR
1732321_5	fabG1_inhA_c-777t (fabG1_c-15t)	rpoB_S450L	fabG1_inhA_c-777t (fabG1_c-15t)	S	S	S	S	S	S	S	S	S	S	MDR	INH+RMP+ETH	INH_MDR
1732565_3	fabG1_inhA_c-777t (fabG1_c-15t)	rpoB_S450L	fabG1_inhA_c-777t (fabG1_c-15t)	S	S	S	S	S	S	S	S	S	S	MDR	INH+RMP+ETH	INH_MDR
1751625_9	katG_S315T	rpoB_S450Y	S	S	S	S	S	S	S	S	S	S	S	MDR	INH+RMP	INH_MDR
1751669_3	katG_S315T	rpoB_S450Y	S	S	S	S	S	S	S	S	S	S	S	MDR	INH+RMP	INH_MDR
MZ168	katG_S315T; fabG1_inhA_c-777t (fabG1_c-15t)	rpoB_S450L	fabG1_inhA_c-777t (fabG1_c-15t)	rrs_A514C	S	embB_M306I	S	S	S	S	S	S	S	MDR	INH+RMP+ETH+SM+EMB	INH_MDR
MZ217	katG_S315T	rpoB_S450L	S	rpsL_K43R	pncA_Y34D	embB_M306I	S	S	S	S	S	S	S	MDR	INH+RMP+SM+PZA+EMB	INH_MDR
MZ252	katG_S315T;fabG1_inhA_t-770a (fabG1_t-8a)	rpoB_S450L	fabG1_inhA_t-770a (fabG1_t-8a)	S	pncA_390_del_1_ca_c	embB_M306I	S	S	S	S	S	S	S	MDR	INH+RMP+ETH+PZA+EMB	INH_MDR
MZ259	katG_S315T; fabG1_inhA_t-770a (fabG1_t-8a)	rpoB_S450L	fabG1_inhA_t-770a (fabG1_t-8a)	S	pncA_V130G; pncA_390_del_1_ca_c	embB_M306I	S	S	S	S	S	S	S	MDR	INH+RMP+ETH+PZA+EMB	INH_MDR
MZ73	katG_S315T	rpoB_S450L	S	S	S	S	S	S	S	S	S	S	S	MDR	INH+RMP	INH_MDR
MZ75	S	S	S	S	S	S	S	S	tlyA_Q22!	S	S	S	S	mono-CPR		
1705390_7	S	S	S	S	S	embB_G406D	S	S	S	S	S	S	S	mono-EMB		
1752035_5	S	S	S	S	S	embB_G406D	S	S	S	S	S	S	S	mono-EMB		
1694395_7	katG_1433_del_1_gc_g	S	S	S	S	S	S	S	S	S	S	S	S	mono-INH		INH_noMDR

1720359_3	katG_S315T	S	S	S	S	S	S	S	S	S	S	S	S	mono-INH		INH_noMDR
1763703_9	katG_S315T	S	S	S	S	S	S	S	S	S	S	S	S	mono-INH		INH_noMDR
1689465_5	katG_S315T	S	S	S	S	S	S	S	S	S	S	S	S	mono-INH		INH_noMDR
1697067_0	katG_S315T	S	S	S	S	S	S	S	S	S	S	S	S	mono-INH		INH_noMDR
1697105_9	katG_S315T	S	S	S	S	S	S	S	S	S	S	S	S	mono-INH		INH_noMDR
1732294_2	katG_S315T	S	S	S	S	S	S	S	S	S	S	S	S	mono-INH		INH_noMDR
1758247_6	katG_S315T	S	S	S	S	S	S	S	S	S	S	S	S	mono-INH		INH_noMDR
1758503_3	katG_S315T	S	S	S	S	S	S	S	S	S	S	S	S	mono-INH		INH_noMDR
1758574_3	katG_S315T	S	S	S	S	S	S	S	S	S	S	S	S	mono-INH		INH_noMDR
1763303_1	katG_S315T	S	S	S	S	S	S	S	S	S	S	S	S	mono-INH		INH_noMDR
1763485_4	katG_S315T	S	S	S	S	S	S	S	S	S	S	S	S	mono-INH		INH_noMDR
MZ172m	katG_1285_del_1_gc_g	S	S	S	S	S	S	S	S	S	S	S	S	mono-INH		INH_noMDR
MZ4	katG_S315T	S	S	S	S	S	S	S	S	S	S	S	S	mono-INH		INH_noMDR
MZ41m	katG_1285_del_1_gc_g	S	S	S	S	S	S	S	S	S	S	S	S	mono-INH		INH_noMDR
MZ76	katG_1866_del_1_ag_a	S	S	S	S	S	S	S	S	S	S	S	S	mono-INH		INH_noMDR
MZ167m	katG_1285_del_1_gc_g	S	S	S	S	S	S	S	S	S	S	S	S	mono-INH		INH_noMDR
1742132_4	katG_1433_del_1_gc_g	S	S	S	S	S	S	S	S	S	S	S	S	mono-INH		INH_noMDR
1732360_4	katG_S315T	S	S	S	S	S	S	S	S	S	S	S	S	mono-INH		INH_noMDR
1732526_4	katG_S315T	S	S	S	S	S	S	S	S	S	S	S	S	mono-INH		INH_noMDR
1751340_1	S	rpoB_V170F	S	S	S	S	S	S	S	S	S	S	S	mono-RIF		
1763326_0	S	rpoB_V170F	S	S	S	S	S	S	S	S	S	S	S	mono-RIF		
MZ151	S	rpoB_H445Y	S	S	S	S	S	S	S	S	S	S	S	mono-RIF		

**MZ155**	S	rpoB_H445N	S	S	S	S	S	S	S	S	S	S	S	mono-RIF		
**MZ56**	S	rpoB_I491F	S	S	S	S	S	S	S	S	S	S	S	mono-RIF		
**1686674_4**	S	S	S	gid_116_del_1_cg_c	S	S	S	S	S	S	S	S	S	mono-SM		
**1686604_1**	S	S	S	gid_116_del_1_cg_c	S	S	S	S	S	S	S	S	S	mono-SM		
**1763537_0**	S	S	S	gid_116_del_1_cg_c	S	S	S	S	S	S	S	S	S	mono-SM		
**1758762_4new**	S	S	S	gid_116_del_1_cg_c	S	S	S	S	S	S	S	S	S	mono-SM		
**1689473_0**	S	S	S	gid_352_del_1_gc_g	S	S	S	S	S	S	S	S	S	mono-SM		
**MZ233**	S	S	S	rrs_C517T	S	S	S	S	S	S	S	S	S	mono-SM		
**MZ261**	S	S	S	rrs_C517T	S	S	S	S	S	S	S	S	S	mono-SM		
**MZ289**	S	S	S	rrs_C517T	S	S	S	S	S	S	S	S	S	mono-SM		
**MZ91**	S	S	S	rrs_C517T	S	S	S	S	S	S	S	S	S	mono-SM		
**MZ296m**	S	S	S	rrs_C517T	S	S	S	S	S	S	S	S	S	mono-SM		
**1705519_2**	katG_S315T	rpoB_S450L	S	rpsL_K43R; rrs_A514C	pncA_C14R	embB_M306I	S	S	S	gyrA_D94G	gyrA_D94G	S	S	pre-XDR	INH+RMP+SM+PZA+EMB+LEV+MXF	
**1758773_0**	katG_S315T	rpoB_S450L	ethA_861_del_1_gt_g	rpsL_K43R	pncA_T135P	S	rrs_A1401G	rrs_a1401g	rrs_a1401g	gyrA_D94G	gyrA_D94G	S	S	pre-XDR	INH+RMP+ETH+SM+PZA+KAN+AMK+CPR+LEV+MXF	
**MZ176**	S	rpoB_S450L	inhA_S94A	S	pncA_L27P	S	S	S	S	S	S	S	S	poly-R	RMP+ETH+PZA	

Abbreviations of Supplementary Table 2: AMI: amikacin, CAP: capreomycin, INH: isoniazid, RIF: rifampicin, ETH: ethionamide, SM: streptomycin, PZA: pyrazinamide, EMB: ethambutol, LEV: levofloxacin, MXF: moxifloxacin; S: susceptible; red: resistant (with the mutation conferring resistance). **Link to download Supplementary Table 2**

References

1. Nguenha D, Manhiça I, Garcia-Basteiro AL, Cowan J. Tuberculosis in Mozambique: Where Do We Stand? *Current Tropical Medicine Reports* 2018; 5: 264–272.

2. Bernardo EL, Nhampossa T, Clouse K, Carlucci JG, Fernández-Luis S, Fuente-Soro L, Nhacolo A, Sidat M, Naniche D, Moon TD. Patterns of mobility and its impact on retention in care among people living with HIV in the Manhiça District, Mozambique. *PLoS One* 2021; 16: e0250844.

3. Kadura S, King N, Nakhoul M, Zhu H, Theron G, Köser CU, Farhat M. Systematic review of mutations associated with resistance to the new and repurposed Mycobacterium tuberculosis drugs bedaquiline, clofazimine, linezolid, delamanid and pretomanid. *J. Antimicrob. Chemother.* Oxford Academic; 2020; 75: 2031–2043.

4. Roberts LW, Malone KM, Hunt M, Joseph L, Wintringer P, Knaggs J, Crook D, Farhat MR, Iqbal Z, Omar SV. Repeated evolution of bedaquiline resistance in Mycobacterium tuberculosis is driven by truncation of mmpR5 [Internet]. bioRxiv 2022 [cited 2023 Jan 23]. p. 2022.12.08.519610Available from: https://www.biorxiv.org/content/10.1101/2022.12.08.519610v1.

5. Walker TM, Lalor MK, Broda A, Ortega LS, Morgan M, Parker L, Churchill S, Bennett K, Golubchik T, Giess AP, Del Ojo Elias C, Jeffery KJ, Bowler ICJW, Laurenson IF, Barrett A, Drobniewski F, McCarthy ND, Anderson LF, Abubakar I, Thomas HL, Monk P, Smith EG, Walker AS, Crook DW, Peto TEA, Conlon CP. Assessment of Mycobacterium tuberculosis transmission in Oxfordshire, UK, 2007-12, with whole pathogen genome sequences: an observational study. *Lancet Respir Med* 2014; 2: 285–292.

6. Walker TM, Miotto P, Köser CU, Fowler PW, Knaggs J, Iqbal Z, Hunt M, Chindelevitch L, Farhat M, Cirillo DM, Comas I, Posey J, Omar SV, Peto TE, Suresh A, Uplekar S, Laurent S, Colman RE, Nathanson C-M, Zignol M, Walker AS, CRyPTIC Consortium, Seq&Treat Consortium, Crook DW, Ismail N, Rodwell TC. The 2021 WHO catalogue of Mycobacterium tuberculosis complex mutations associated with drug resistance: A genotypic analysis. *Lancet Microbe* 2022; 3: e265–e273.
